# Glycoconjugates in Host-Helminth Interactions

**DOI:** 10.3389/fimmu.2013.00240

**Published:** 2013-08-28

**Authors:** Nina Salinger Prasanphanich, Megan L. Mickum, Jamie Heimburg-Molinaro, Richard D. Cummings

**Affiliations:** ^1^Department of Biochemistry, Glycomics Center of Emory University, Emory University School of Medicine, Atlanta, GA, USA

**Keywords:** glycans, glycoconjugates, helminths, C-type lectin, innate immunity, adaptive immunity, anti-glycan antibodies, schistosomiasis

## Abstract

Helminths are multicellular parasitic worms that comprise a major class of human pathogens and cause an immense amount of suffering worldwide. Helminths possess an abundance of complex and unique glycoconjugates that interact with both the innate and adaptive arms of immunity in definitive and intermediate hosts. These glycoconjugates represent a major untapped reservoir of immunomodulatory compounds, which have the potential to treat autoimmune and inflammatory disorders, and antigenic glycans, which could be exploited as vaccines and diagnostics. This review will survey current knowledge of the interactions between helminth glycans and host immunity and highlight the gaps in our understanding which are relevant to advancing therapeutics, vaccine development, and diagnostics.

## Introduction

Helminths are multicellular parasitic worms that comprise a major class of human pathogens. They rely on a host species to complete a portion of their life cycle, which results in significant morbidity for human and animal hosts. The three classes of helminths – nematodes, trematodes, and cestodes – account for half of the WHO-designated “Neglected Tropical Diseases,” and infect 1–2 billion of the world’s poorest people, with soil-transmitted helminths (gastrointestinal nematodes including *Ascaris*, *Trichuris*, *Necator* sp.) and schistosomes (blood-dwelling trematodes) being the most common (1–3). Although great strides have been made through implementation of chemotherapy and improved sanitation, massive amounts of suffering due to helminth infections persist, and to date, no vaccines for helminths or any human parasite exist.

The symptoms of helminth infection depend on infection intensity (i.e., number of worms and/or eggs), and range from none to chronic disease, disfigurement, and death. The majority of cases in endemic regions manifest with low-level symptoms such as anemia, malnutrition, and delayed physical/cognitive development (1, 4). The estimated disease burden of helminth infection is at least 13,000,000 DALYs (years of life and productive life lost due to disability and/or death) (2). However, this is probably a gross underestimation of the total disease burden because several common helminth infections are excluded, and DALYs fail to account for the social and economic consequences caused by the subtler symptoms mentioned above (1, 5, 6). Some estimates therefore rank the burden of helminth infection even higher than that of malaria or HIV/AIDS, making helminths a true “societal poverty trap” (4, 7, 8).

While a substantial body of literature on the biology and immunology of helminth infection exists, the science has yet to translate into more sophisticated solutions for diagnosis, treatment, or prevention. This stems from a poor understanding of protective immunological mechanisms, insufficient knowledge of unique molecular structures of helminths, and a lack of innovative vaccine strategies to protect against complex, multicellular pathogens. The complex carbohydrates of helminths present an exciting opportunity to fill these gaps. Many glycans within glycoproteins and glycolipids are unique to helminths or to a particular worm species, they are abundant on worm surfaces and secretions, and humans vigorously target these glycans in the natural immune response. Helminth glycans also have potent immunomodulatory effects. Advances in glyco-technology have steadily increased our ability to understand this often-overlooked area of host-pathogen interactions. In this review, we will discuss the role of carbohydrates in helminth innate and adaptive immunity, highlight glycan structures of interest, and call attention to progress in exploiting these structures for modulation of autoimmune/atopic diseases and better control of helminth infection.

### The interface of helminths and their hosts

Each human helminth has a complex, multi-stage lifecycle, which depends on particular intermediate and definitive hosts and an ecological niche. For example, *S. mansoni*, the most common cause of schistosomiasis, lives only in fresh water inhabited by the mollusk host *Biomphalaria glabrata*. Its eggs hatch into miracidia which penetrate susceptible snails. The miracidia circulate in the snail hemolymph and transform into sporocysts, which over the course of about a month generate free-swimming cercariae that exit the snail (9, 10). Cercariae penetrate the skin of a human host or other mammals exposed to water harboring infected snails. In the process, they are transformed into schistosomula larvae, which, after a few days in the dermis, make their way into the venous circulation. Within 1–3 weeks they traverse the narrow pulmonary capillary beds and move to the portal vessels, feeding on blood, and growing in size as they move. Male and female worm pairs mate and migrate up the mesenteric vein, where they commence egg laying, about 5–6 weeks after initial infection. Eggs excreted into the stool continue the schistosome life cycle if they are deposited back into fresh water, while others become trapped in the intestinal walls and liver (11). Other mammals are also infected by schistosomes and serve as major reservoirs of transmission (12). Hookworms, by contrast, such as *Necator americanus* and *Ancylostoma duodenale*, have only humans as their definitive host (4). The larvae live freely in the soil for a short period of time while they develop to the L3 stage larvae, which, like schistosomes, penetrate the skin, and migrate into the vasculature. Upon reaching the lungs, they migrate up to the pharynx, at which point they are swallowed. The larvae molt, and male and female adult worms embed in the mucosa and submucosa of the intestines in order to mate and feed on blood (7, 9). Helminths can live for years to decades in a human host, continuously producing eggs.

The helminth’s interface with host immunity is equally complicated. Nematodes are protected by a layer of collagen that comprises the cuticle, which is overlain by a lipid-rich epicuticle and a glycoprotein surface coat. The cuticle is re-synthesized and shed every time the worm enters a new developmental stage (13). The surface of the schistosome is complex, incompletely understood, and variable throughout its life stages. The outer layer consists of a tegument, a syncytial layer of cells which are bounded apically by a complex invaginated membrane (14). The tegument is comprised of secreted lipid-rich “membranocalyx,” as well as “glycocalyx,” the latter of which is partially discarded upon transformation of cercariae to schistosomules, but also appears to be prominent on the surface of adult worms (15–18). The expression of both proteins and glycans is regulated from one life stage to the next, and highly variable (19, 20). The surface of the worms as well as excreted and secreted products, molted tissue layers, and eggs make up the targets for immune recognition and attack. The gastrointestinal (GI) tract of blood-feeding worms like schistosomes and hookworms is also exposed to antibodies (21, 22).

## Glycans of Schistosomes and Other Parasitic Helminths

Parasitic helminths are characterized by their production of many different glycoproteins, containing complex *N*- and *O*-glycans, and glycolipids; all of these glycans are unusual and structurally distinct from host glycans (some are depicted in Table 1). For example, helminths, such as *S. mansoni*, neither synthesize sialic acid nor acquire it from their hosts, whose glycans typically terminate in sialic acid (23). Helminth glycans commonly terminate with β-linked GalNAc (24–27), often in the sequence GalNAcβ1-4GlcNAc (termed the LacdiNAc motif, LDN), which is not commonly present in vertebrate glycans (28, 29). In addition, many helminths use unusual sugars, such as tyvelose, found in *N*-glycans of *Trichinella spiralis* (30–32), which may be useful in both resistance to infection (33) and diagnostics (34, 35). Several helminths also generate unusual modifications of sugars, such as the phosphorylcholine (PC) modification of glycans of *Echinococcus granulosus*, several other parasitic nematodes, and the free-living *Caenorhabditis elegans* [(36–41); and reviewed in (42)], and 2-O-methylation of fucose and 4-O-methylation of galactose in highly antigenic glycans of *T. canis* (43, 44). In *S. mansoni* glycans, unique additions of fucose residues are seen on both GlcNAc and GalNAc residues in the LDN motif, giving rise to FLDN, LDNF, poly-LDNF, DF-LDN-DF (27, 45–51), as well as unique fucose/xylose modifications of the *N*-glycan core (23, 52, 53) (Table 1). Some nematodes, of which *C. elegans* is best studied, also oddly modify their core fucose residues with galactose (54–58). Interestingly, only the trematode *S. mansoni* (59–61) and the cattle lungworm nematode *Dictyocaulus viviparous* (62) have been shown to synthesize glycans containing the terminal motif of the Lewis x (Lex) antigen, variants of which are also expressed commonly on human cells (61, 63). Schistosomes synthesize novel glucuronate-containing glycans on glycoproteins, such as the CAA structure (64, 65). The core structures of the glycolipids in helminths are also unlike those of mammals, such as the presence of the “schisto motif” GalNAcβ1-4Glcβ-Cer (25) of *S. mansoni*, and the “arthro motif” Manβ1-4Glcβ-Cer of *A. suum* (66), instead of the mammalian “lacto motif” Galβ1-4Glcβ-Cer.

**Table 1:** **A selection of helminth glycan structures involved in innate and adaptive immunity**. 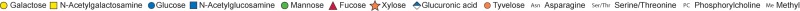

Glycan	Structure	Species	Antigenic?	Receptors	Effects/functions	Reference
LN Lewis x (Lex)	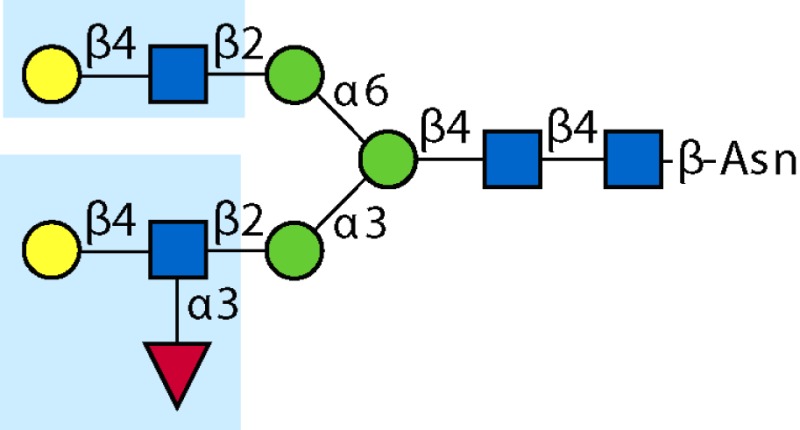	S. mansoni* S. mansoni*	No Yes	DC-SIGN, MR (weak)	Granuloma induction B cell proliferation, IL-10 production, TH2 bias, immunosuppressive, antibodies can mediate complement lysis of leukocytes	van de Vijver et al. (227) Srivatsan et al. (59), Velupillai and Harn (102), Nyame et al. (63), van Die et al. (117), Meevissen et al. (120)

Poly-Lex	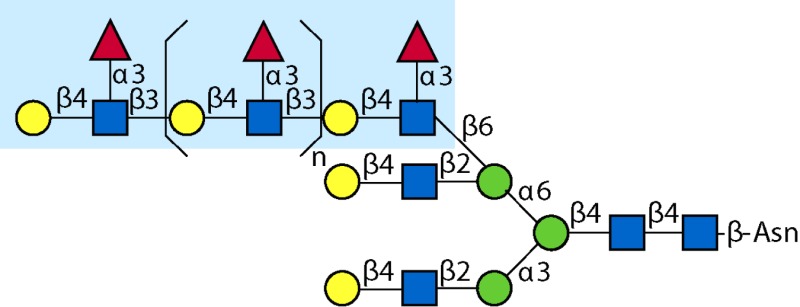	S. mansoni*	Yes			Srivatsan et al. (59), van Roon et al. (228), Mandalasi et al. (60)

LDN LDNF	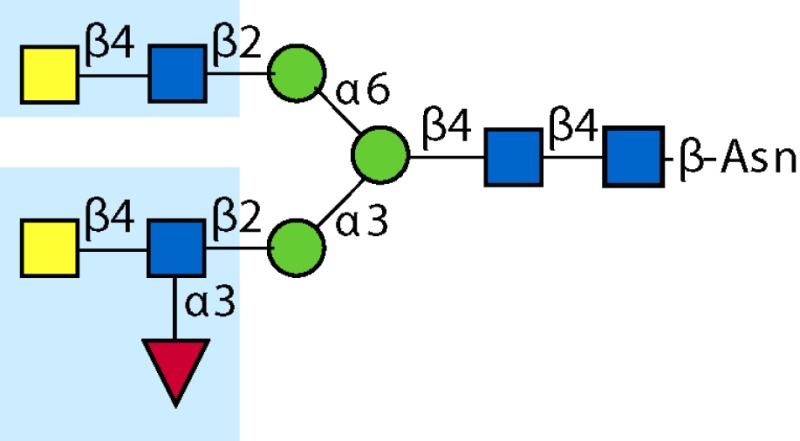	S. mansoni* Schistosoma spp.*, *H. contortus*, *T. spiralis*	Yes Yes	MGL; galectin-3 DC-SIGN; MGL; MR; CD62E (E-selectin)	Granuloma induction; possible molecular mimicry with snail host; antibodies to LDN lyse schistosomula *in vitro* DC maturation; antibodies correlate with protection to *H. contortus*	Srivatsan et al. (27), Neeleman et al. (28), Nyame et al. (122, 224), van den Berg et al. (100), van Vliet et al. (119), van de Vijver et al. (227), Meevissen et al. (120), Yoshino et al. (284) Srivatsan et al. (27), Nyame et al. (223), van Die et al. (117), van Vliet et al. (119), van Liempt et al. (118), Meevissen et al. (120), van Stijn et al. (128)

Poly-LDNF	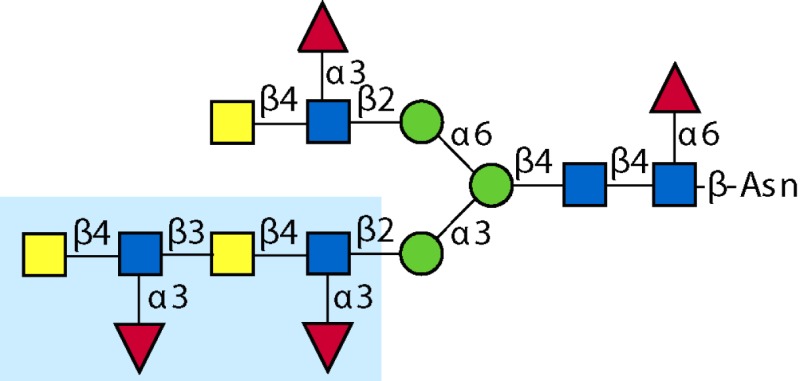	*S. mansoni*	Yes	DC-SIGN		Kawar et al. (289), van Liempt et al. (118), Wuhrer et al. (48, 229)

FLDN LDN-DF	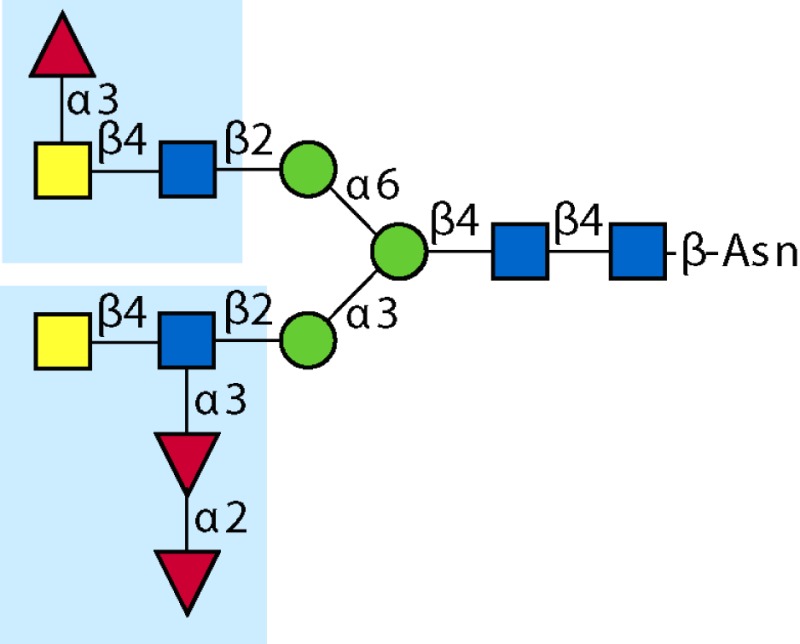	*S. mansoni* *S. mansoni*	Yes Yes	DC-SIGN	Stimulates IL-10, IL-6, and TNF-α production by PBMC	Naus et al. (234), van Remoortere et al. (233), de Boer et al. (236), Meevissen et al. (120), Frank et al. (51) van der Kleij et al. (116), Naus et al. (234), Frank et al. (51), van Remoortere et al. (233)

FLDNF	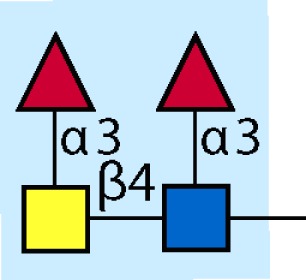	*S. mansoni*	Yes		Antibodies to FLDNF are protective in rats	Geyer et al. (50), Grzych et al. (171, 221, 222), Wuhrer et al. (46), Kantelhardt et al. (49)

DF-LDN-DF	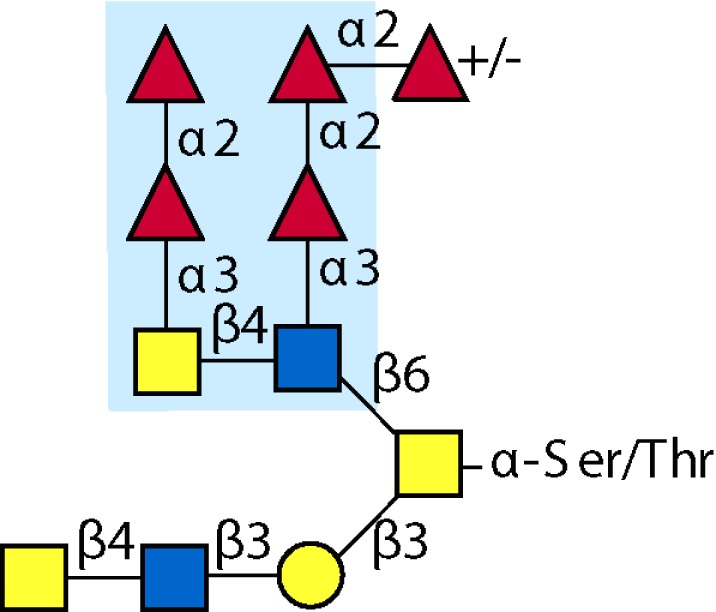	*S. mansoni*	Yes		Diagnostic in urine	Robijn et al. (243, 250)

Core β2 Xyl, core α3 Fuc	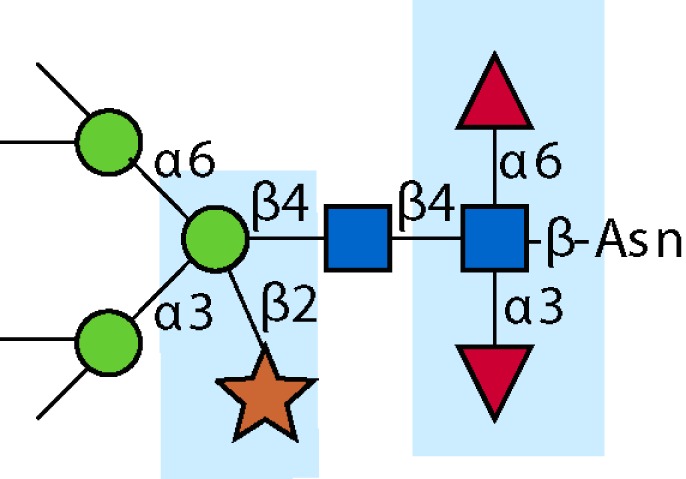	*S. mansoni*	Yes		Th2 biasing of DCs	Faveeuw et al. (114), Meevissen et al. (120)

Circulating cathodic antigen (CCA)	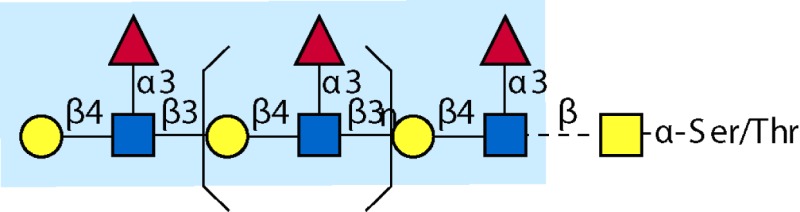	*S. mansoni*	Yes		Diagnostic (urine, sera)	Deelder et al. (65), van Dam et al. (245)
Circulating anodic antigen (CAA)	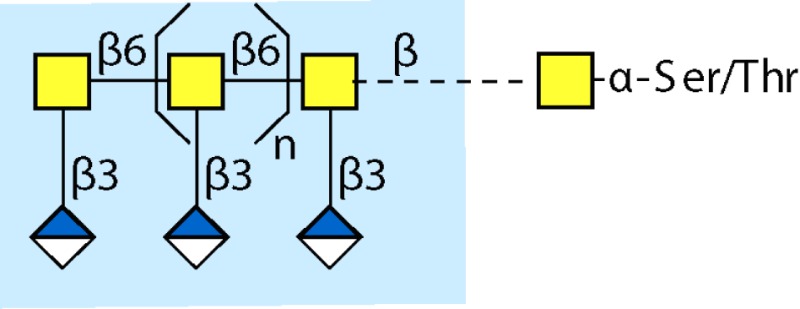	*S. mansoni*	Yes		Diagnostic (urine, sera), forms antibody-antigen complexes	Deelder et al. (65), Vermeer et al. (244)

Tyvelose	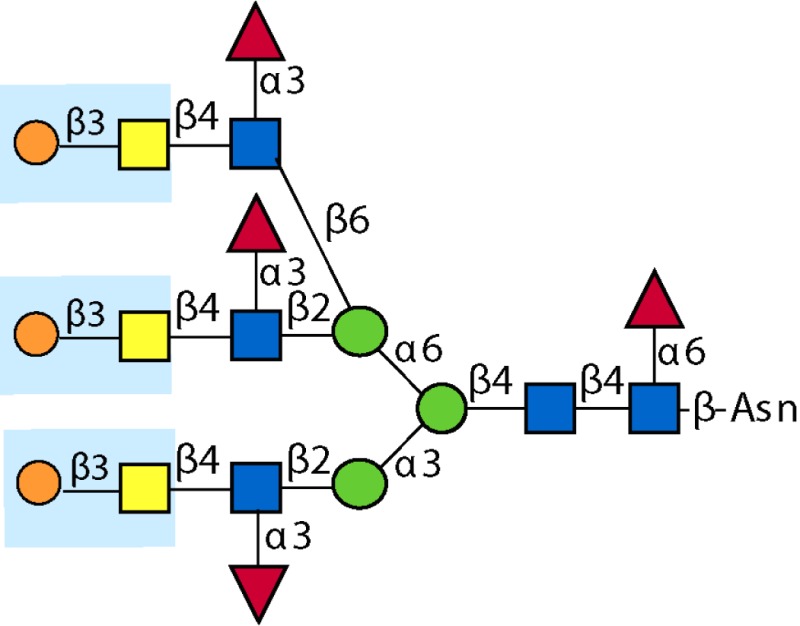	*T. spiralis*	Yes		Antibodies to tyvelose are protective and diagnostic	Ellis et al. (30), Reason et al. (31), McVay et al. (215), Bolás-Fernandez and Corral Bezara (72)

Galα1-3GalNAc	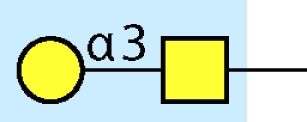	*H. contortus*	Yes		Antibodies to Galα1-3GalNAc are protective	van Stijn et al. (257)

Gal-Fuc	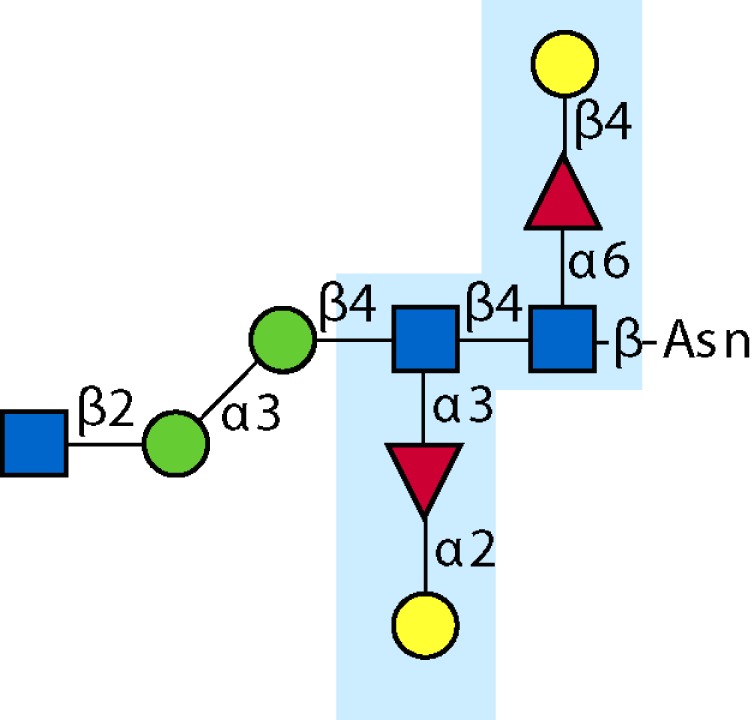	*C. elegans*, *A. suum*	Yes	Endogenous and fungal galectins; human Gal-1	Fungal CGL2 kills *C. elegans*	Yan et al. (54), Butschi et al. (80), Takeuchi et al. (57, 58)

Galα1-4Galβ1-3GalNAc	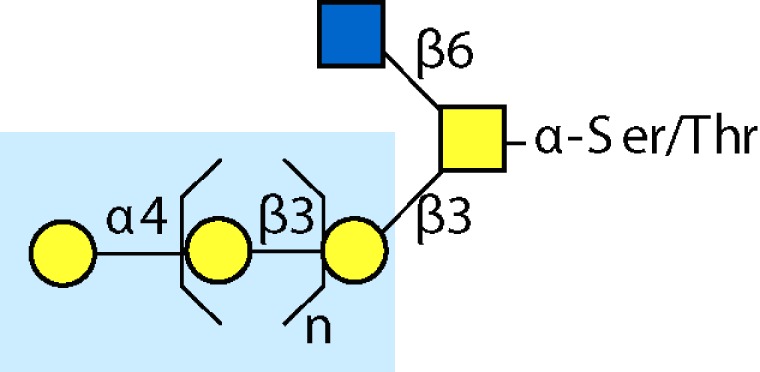	*Echinococcus* spp.	Yes		Diagnostic	Koizumi et al. (254), Díaz et al. (255)

PC-glycan	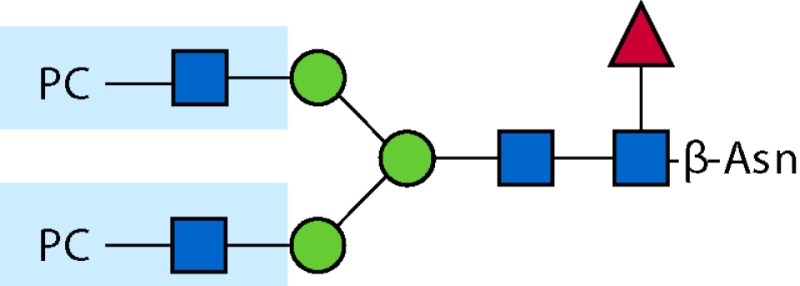	Filarial and GI nematodes, *E. granulosus*	Yes		Anti-inflammatory (both Th1 and Th2); nematode development	Fletcher et al. (41), Peters et al. (217), Paschinger et al. (37), Rzepecka et al. (142), Grabitzki et al. (39)

Methylated Fuc/Gal	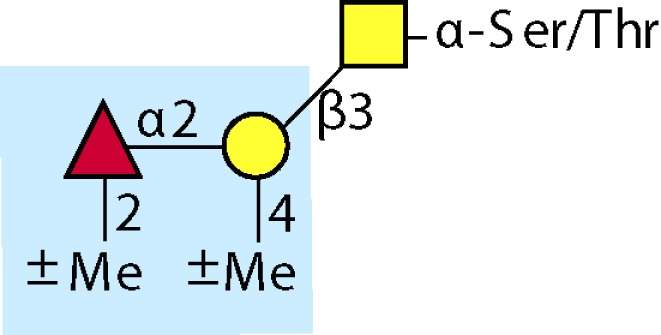	*Toxocara* spp.	Yes			Khoo et al. (43), Koizumi et al. (44)

PC-glycolipids	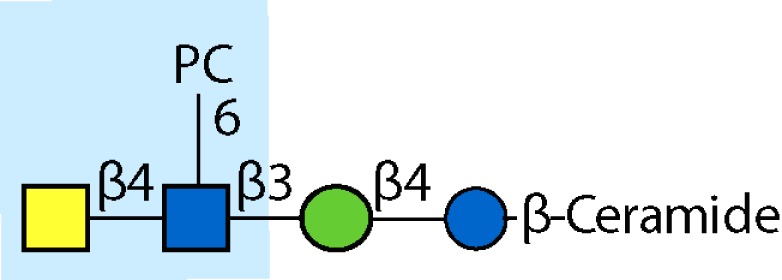	*Ascaris* spp.	Yes		Inhibits LPS-induced B cell proliferation and macrophage, IL-12 release; induces PBMC to produce Th1 cytokines	Lochnit et al. (132), Deehan et al. (133), van Riet et al. (134)

*Defined determinants (common names are indicated) recognized by either antibodies or glycan-binding proteins and lectins are indicated in the blue background boxes, along with the known helminths expressing the determinant. Note that many structures are composite examples, and that some complex and branched glycans may possess one or more of these determinants, such as outer branch LDN-DF and inner core Xyl and Fuc residues. The asterisk indicates glycan motifs that can also be made in mammalian hosts, but are not necessarily cross reactive, due to differences in surrounding structures. Their antigenicity is also noted if they have been confirmed as antibody targets, as well as any known receptors and demonstrated functions, *in vitro* or *in vivo*, with selected corresponding references*.

The unusual nature and antigenicity of parasitic helminth glycans belies the apparently commonly held belief among immunologists and parasitologists that parasites do not express antigenic glycans, but rather cloak themselves in parasite-synthesized and/or host-acquired antigens to avoid immune recognition in what has been termed “molecular mimicry” or “antigen sharing” (67, 68). This concept may no longer be tenable as a general description in regard to parasitic helminths, which synthesize few glycans resembling their vertebrate hosts. In fact, glycans constitute a major portion of the host’s antigenic targets in several helminth infections. In non-human primate models of schistosomiasis, they appear to be even more highly targeted than proteins (69–74). When true molecular mimicry by infectious organisms does occur, such as the structural similarity between mammalian ganglioside GM1 and the terminal structure of the lipooligosaccharide from *Campylobacter jejuni*, the mimicry is associated with pathological autoimmunity, as seen in Guillain–Barré syndrome (75, 76). Interestingly, few of the antibodies to helminth glycans cross-react with host glycans. The only well-known example of this is Lex (63), suggesting that even helminth glycans sharing some features with rare mammalian glycoconjugates, such as LDN and LDNF (Table 1) are presented in a unique fashion on parasites. As discussed below, results of multiple studies indicate that parasites instead utilize “glycan gimmickry” (77), in which their glycans can interact with host receptors to modulate host immune responses to the benefit of the parasite.

## Innate Immune Responses to Helminth-Derived Glycans

The response of an infected host to a parasitic helminth is multifaceted and involves both innate and adaptive immune factors, and a host of cellular responses. While this review section is devoted to mammalian responses to helminth infections, it is worth noting that other organisms have also developed a wide range of responses to helminth infections. For example, one lectin (CNL) from the mushroom *Clitocybe nebularis* (78) can bind to the LDN motif, and the recombinant form of the CNL can directly kill the hypersensitive *C. elegans* mutant strain pmk-1 (79). Other types of fungi, on which *C. elegans* feeds, express specific lectins that recognize the core galactose-fucose determinants (57). These are highly expressed on the worm’s intestinal cells, and ingestion of such fungi causes death of the nematode (80). The zebrafish (*Danio rerio*) is parasitized by the pathogenic nematode *Pseudocapillaria tomentosa* (81). Similarly to human gastrointestinal worms, infection causes eosinophilic inflammation in the fish gut, offering a potentially promising new model with which to understand the interactions between helminth ligands and host innate immune receptors (82, 83).

Mammalian immune responses to parasitic helminths are incredibly complex. In some mammalian hosts, the adaptive response may help to prevent, limit, or eradicate the infection, while in others it appears ineffectual (84). The ability of adaptive effector mechanisms to limit or clear infection likely depends, in large part, on cues received from the innate response. The innate response can both limit the pathology of the infection and directly contribute to destruction and expulsion of worms. However, the parasites have evolved glycan gimmickry approaches to battle the host responses. Thus, the balance arrived at in a chronic infection may result in asymptomatic infection even though humans rarely clear all of the infecting organisms without treatment (84, 85).

**Figure 1:**
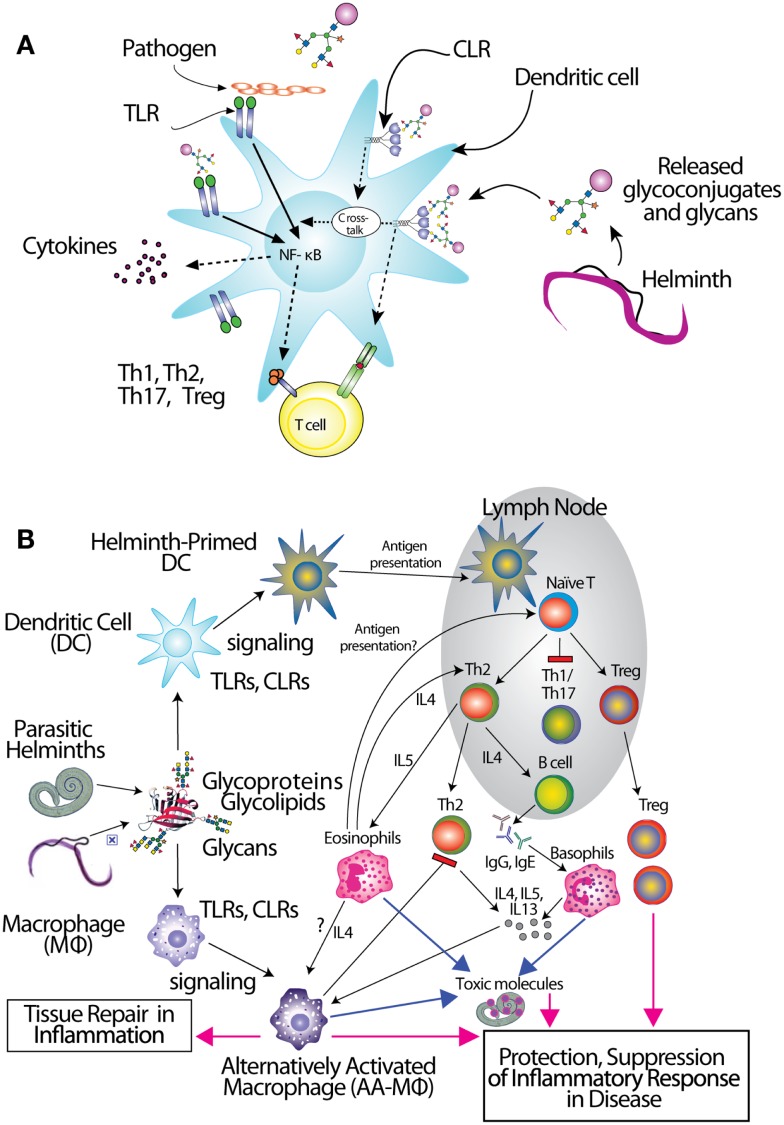
**Interactions of glycans with immune cells and regulatory pathways**. **(A)** Glycan-binding proteins such as the C-type lectin receptor (CLR) DC-SIGN in cooperation with Toll-like receptors (TLR), such as TLR4, regulate dendritic cell responses to parasite glycans. **(B)** Schematic representation of the role of dendritic cells (DC) and macrophages (MΦ) in inducing an anti-inflammatory adaptive immune response upon contact with helminth glycans.

Antigen-presenting cells (APCs) including dendritic cells (DCs) and macrophages (MΦ) initially encounter invading pathogens and are crucial for regulation of the type of adaptive immune response (86, 87) (Figure 1). Helminths induce effector cell generation consisting of Th2, T regulatory cells (T_regs_), and alternatively activated (AA) MΦ (88–91), which may contribute to the capacity of helminths to counteract inflammation associated with autoimmune disease. Recognition of pathogen glycans is known to be mediated by at least two classes of specialized pattern-recognition receptors (PRRs) on APC, the Toll-like receptors (TLRs) and C-type lectin receptors (CLRs), which are instrumental in regulation of adaptive immunity (92–94). There are over a dozen different C-type lectins expressed in DC and Langerhans cells, and many other glycan-binding proteins, such as selectins, siglecs, and galectins expressed by lymphocytes, all of which have potential to interact with parasite-derived glycans (95, 96). TLRs function as PRRs that can recognize a wide variety of foreign molecular patterns (pathogen-associated molecular patterns or PAMPs), as seen, for example, where they recognize the many variants of LPS. While CLRs can also function as PRRs, their specificity is often much more restricted, as seen with dectin-1, which is a receptor for β-glucan (97). The balance between CLR- and TLR-mediated signals appears crucial to determine the balance between tolerance and immunity (92, 98, 99). Human galectins-1 and -3 have been shown to recognize the core galactosylated-fucose epitope that is expressed in nematodes and the LDN-motifs that are common in schistosomes, respectively, implying a role for galectins in pattern recognition of parasitic helminths (58, 100).

Little has been done on direct effects of intact worms on APC, and the mechanistic roles of glycans in glycan gimmickry, but several studies using soluble extracts of worms or their eggs have demonstrated the importance of helminth glycans in immunomodulation. Early observations showed that egg deposition was responsible for the Th2 character of chronic murine schistosomiasis (101). The Harn group followed up on these observations by showing that LNFPIII, a human milk sugar containing Lex, induced B cell proliferation and IL-10 production by murine spleen cells (102). They also demonstrated that intranasal administration of *S. mansoni* soluble egg antigen (SEA) extracts to mice promoted IgE and IgG1 production and induced secretion of IL-4, IL-5, and IL-10, but not IFN-γ, by lymphocytes (103, 104). These responses were completely dependent on the presence of intact helminth glycans, since partial oxidation of glycans with periodate abolished the ability of SEA to stimulate these Th2 responses (103). Both SEA and soluble worm proteins from *Trichuris suis* (TSWAP) inhibit LPS-induced secretion of many pro-inflammatory cytokines and chemokines from DC (99, 103, 105). This suppressive effect was also periodate-sensitive, while protein denaturation at 80°C, and digestion of the glycoproteins with chymotrypsin had no effect (106). In addition, co-incubation of immature DC with LPS and helminth compounds induced a decrease of CD86 surface expression (99) and a strong upregulation of OX40L expression on the DC surface which was glycan-dependent (105, 106). Other studies showed that immunization of mice with soluble extracts of many different helminths, including *C. elegans*, the roundworm *Brugia malayi*, and the tapeworm *Taenia crassiceps*, also induced a glycan-dependent cytokine response biased toward Th2 cells (107–109). One of the glycan determinants which contributes to the Th2-biasing effect of SEA is Lex (103, 110–113), but other schistosome glycans can also induce Th2 biasing, such as core fucosylated/xylosylated *N*-glycans (114). The unique abilities of helminth glycolipids to drive Th2 bias may involve CD1d-restricted T cells (115). Treatment of monocytes with *S. mansoni* egg glycolipids, but not adult worm glycolipids, stimulated IL-10, IL-6, and TNF-α production, which was largely dependent on expression of the LDN-DF motif, indicating that helminth glycolipids can induce both pro- and anti-inflammatory cytokine secretion (116).

In regard to the mechanisms of glycan recognition, several CLRs of DC and MΦ, such as DC-SIGN, bind selected glycans, including Lex, LDNF, and poly-LDNF (117, 118) on the defined glycan microarray from the Consortium for Functional Glycomics (CFG). Human MΦ galactose-type lectin (MGL), expressed as an Fc fusion protein, binds to a subset of glycans on the CFG microarray, with highest recognition of those containing terminal GalNAc residues (119). Related studies using similar microarray approaches have also defined specific interactions of DC-SIGN, mannose receptor (MR), and MGL with schistosome-related glycans containing Lex motifs, LDN, LDNF, as well as core β2Xyl glycans (120). MGL is selectively expressed on APC with elevated levels on tolerogenic DC and AA-MΦ (121), suggesting a role of MGL in the homeostatic control of adaptive immunity. This is consistent with earlier studies showing that DC-SIGN binds components within SEA of *S. mansoni*, as do the CLRs MR and MGL (99). SEA expresses many of the fucosylated glycans used in the microarray studies above. In particular, LDNF and Lex antigens are expressed on all intra-mammalian stages of the parasite (27, 59, 122). We also confirmed the differential binding profile of DC-SIGN and MGL to SEA and TSWAP by ELISA (106).

The CLRs mentioned above induce endocytosis of bound molecules for antigen presentation but do not induce classical signs of APC activation. They do, however, modulate the gene transcription induced by other receptors (Figure 1A), such as NF-κB signaling downstream of TLRs (123). Interestingly, there is evidence that TLR4 may be involved in responses to *S. mansoni* Lex-containing glycans (111), indicating interactions and co-signaling via TLR and CLR may contribute to the overall polarization of immunosuppressive responses to the parasite infections. Recent studies in DC reveal the capacity of some CLRs to induce intracellular signaling cascades upon binding to pathogen-derived glycans, and show that CLR-induced signals intersect with the signaling pathways of several TLRs, including TLR2, TLR4, and TLR8. CLR signaling can “override” the response to a variety of otherwise pro-inflammatory TLR ligands such as LPS, instead inducing secretion of Th2-type or immunoregulatory cytokines, in a TLR-specific manner (124–127). In contrast, *S. mansoni* fucosylated glycolipids induce a pro-inflammatory response in DCs that is dependent on both DC-SIGN and TLR4 (128). The specific signaling interactions which contribute to this diverse response modulation are still being explored. Novel roles for CLRs interacting with schistosome glycoconjugates have been suggested by a glycoform of RNAse termed omega-1 (129), where uptake by MR may contribute to RNAse internalization and impaired protein synthesis through degradation of both ribosomal and messenger RNA (130).

Thus, while much remains to be learned about parasite glycans and their bioactivities, the glycans of parasitic helminths have unique functions in innate immune responses and induce both CLR signaling as well as cross talk with TLR signaling in the human system. The molecular mechanisms of glycan-dependent innate immune responses are also linked to the adaptive immune responses, as discussed below. Understanding these responses could well lead to the development of novel therapeutic glycans that could be useful in treating human diseases associated with inflammation and autoimmunity.

### Applications of helminth glycans to other inflammatory and infectious diseases

The immunomodulatory properties of helminth glycans are relevant not only to the outcomes of helminth infections, but may also be relevant to the outcomes of vaccinations, co-infections, and inflammatory disorders. Recently, many investigators have focused on understanding the effect that helminth immunomodulation has on responses to co-endemic infections such as *Mycobacterium tuberculosis* (MTB). Interestingly, it was found that *N. brasiliensis*, a mouse model for GI helminth infection that passes through the lungs, impairs ability to control MTB infection, and that this effect was mediated by IL-4 signaling of alternatively activated (M2 type) macrophages (131). Little work has been done on *N. brasiliensis* glycoconjugates, but they do have Tn antigen, and PC-containing glycoconjugates, which have several anti-inflammatory and other immunomodulatory actions (42, 132–135).

Helminth infections and their products have a phenomenal ability to ameliorate responses to a variety of inflammatory disorders (136). For example, in clinical studies of patients suffering from inflammatory bowel disease, treatment with the pig nematode *T. suis*, caused remission of Crohn’s disease for more than half of the patients and improved the symptoms of Ulcerative Colitis for many patients (137, 138). Recently, two small clinical trials of multiple sclerosis (MS) patients – one comparing uninfected to those with naturally acquired *T. suis* infection, and the other using *T. suis* ova as treatment – suggested that *T. suis* may decrease unfavorable MRI changes, reduce exacerbations, and results in favorable immunological parameters such as elevated IL-4 and IL-10 (139–141). The PC-containing helminth product ES-62 was recently shown to protect against airway inflammation in a mouse model of asthma (142).

Studies of helminth anti-inflammatory effects on some other disorders have been less favorable, such as the use of the hookworm *N. americanus* in Celiac Disease patients and *T. suis* for allergic rhinitis (143–145). The reasons for these failures are still unclear, but may include insufficient dose of worms, provocation of a mixed rather than purely immunoregulatory cytokine profile at safe doses, or a lack of effect at the level of symptoms even when the desired immunosuppressive responses are achieved in response to helminth treatment (146, 147). While controlled ingestion of therapeutic helminths has thus far been safe for adults, it can also cause significant gastrointestinal side effects (148). The anti-inflammatory molecules produced by the parasites, many of which, as mentioned above, are glycoconjugates, are not yet well defined. A better understanding of these molecules would allow us to channel the immunomodulatory properties of helminths into purer and more potent immunoregulatory therapies, with great potential for treating multiple chronic inflammatory diseases.

## Adaptive Immune Responses to Helminth-Derived Glycans

Helminth infections present a dual challenge to immunologists: Firstly, we have an insufficient understanding of the immune effector mechanisms that successfully combat worms. Secondly, the study of adaptive immunity to eukaryotic pathogens has traditionally focused on protein, rather than glycan antigens. A large portion of the surface-exposed and secreted antigens of helminths consists of glycoconjugates (17, 149, 150). Thus, crucial insights into immunological control of helminth infection lie at the intersection of these two fields, as we will now discuss.

### The character of adaptive immunity to helminths

Due to the immunomodulatory effects of several glycoconjugates mentioned above, helminths usually elicit a Th2 response (Figure 1B). Non-endemic individuals newly exposed to *S. mansoni* can suffer from a more Th1-type acute disease known as Katayama fever, in which elevated levels of TNF, IL-1, and IL-6, accompany eosinophilia (11, 151), but people in endemic regions rarely suffer acute symptoms. Instead, they seem to be pre-disposed to developing a chronic, Th2-type response, the onset of which coincides with egg laying (11). This may stem from sensitization *in utero* or very early in life (152). The immune response to chronic helminth infection is dominated by a self-reinforcing Th2 feedback loop between cytokines IL-4, IL-5, IL-13, and prominent expansion of eosinophils and mast cells (8, 84). Initiation of this Th2 feedback loop has been a topic of intense investigation in the last few years, implicating mast cells, basophils, eosinophils, alternatively activated macrophages, and epithelial cells, just to name a few, as being required to initiate production of Th2 cytokines. Most recently, novel innate immune cell types such as the nuocyte have surfaced as the most likely Th2-initiating cells [reviewed in (153)]. Whatever the initiating Th2 cell type(s), it is likely that they receive important signals from helminth glycoconjugates, and little work has been done on the interactions of these molecules with such “unconventional” Th2-initiators.

During the chronic Th2 response, abundant antibodies of all subtypes are produced, especially IgE, IgG1, and IgG4 (22, 85, 154). In schistosomiasis, chronic pathology is primarily due to eosinophilic (type 2) granulomas, consisting of macrophages, CD4^+^ T cells, eosinophils, and collagen that surround eggs trapped in liver, intestinal, or bladder tissue, which are eventually converted to fibrotic scars (11, 155). Many other nematodes and cestodes also cause eosinophilic granulomas (84, 156–158).

The regulatory response is crucial in control of chronic helminth disease, for the well-being of both host and parasite (Figure 1B). Schistosomes, hookworms, and filarial nematodes all promote the development of T_regs_, and the production of regulatory cytokines like IL-10 and TGF-β from multiple cell types, and IgG4, a non-complement fixing isotype. This type of response, collectively termed “modified Th2,” serves to limit the immunopathology that would result from an uncontrolled Th2 amplification-loop, and allows the host to remain relatively healthy for the long duration of helminth infection (84, 159, 160). In concordance with this idea, schistosomiasis patients with chronic liver and spleen inflammation lack the IL-10 response to worm antigens, which is observed in chronic patients with low-level symptoms (85). AA-MΦ also aid in limiting worm-induced immunopathology. Alternative activation of macrophages is induced by Th2 cytokines like IL-4 and IL-13 as well as directly by the products of several helminths, including *S. mansoni*, *F. hepatica*, filarial nematodes, and tapeworms (90, 150, 161–164). Though we have only just begun to define the sequelae of helminth glycoconjugate interactions with innate immune receptors, described above, it seems likely that this class of molecules plays a large role in dictating the character of the immune response to infection.

### Correlates of protection from helminth infection

Although the association of Th2-type immunity with helminths has been recognized for decades, we are still unraveling the effector mechanisms through which Th2 components control worm infections. Animal infections with gastrointestinal nematodes provide a model of an effective Th2-mediated response. Immunity to intestinal nematodes depends on Th2 cytokines (IL-4, IL-5, IL-9, and IL-13) and is antagonized by Th1 cytokines. Mast cells and basophils are critical for expulsion of GI worms in some animal models, but are not always necessary (165, 166). Th2 cytokines have important protective effects directly on epithelial cells, including goblet cell hyperplasia, increased smooth muscle contractility, and secretion of molecules that directly target worms (22, 164, 165).

Animal models of helminth infection have demonstrated that some immunological effector mechanisms are successful in combatting helminth infection. In the brown rat, which eliminates *S. mansoni* before patency, complement fixation, IgG2a and IgE levels, mast cell degranulation, and eosinophil-mediated antibody-dependent cellular cytotoxicity (ADCC) have been cited in protection (167–172). In rhesus macaques, another protective model for schistosomiasis where adult worms become attenuated in the weeks after reaching patency, IgG-mediated complement killing of schistosomules, and neutralization of adult worms have been demonstrated (173–175). Other animal models have shown that eosinophils, monocytes/macrophages, and neutrophils can mediate *in vitro* ADCC of various helminth larvae including *S. mansoni*, *F. hepatica*, and *S. stercoralis* (176–178).

In human schistosomiasis cohorts, some adults acquire fewer infections and have lower worm burdens compared to children and more susceptible adults (179). Eosinophilia is a relatively well-established correlate of human schistosomiasis resistance (180, 181). Human eosinophils can kill schistosomula *in vitro* via IgG from infection antisera (182–184), however, there is no direct evidence that ADCC occurs during the course of human or animal infection, and eosinophilia can also be accounted for by the presence of type 2 granulomas (185). Mouse models of eosinophil knockout and eosinophil depletion have implicated a protective role for this cell type in some tissue-dwelling nematodes, but for many helminth models, eosinophils appear to play no role in protection [(186); and reviewed in (187)]. Rather than playing a direct role in the damage of worms, their importance may be to support other cells which have been shown to act directly on worms, such as basophils and alternatively activated macrophages [(188); and reviewed in (189)]. Thus, whether eosinophils contribute to protection in human helminth infections remains controversial.

High IgE levels (to heterogeneous schistosomula and adult antigens, as well as more specific antigens, such as Sm22) and high IgE/IgG4 ratios, are well-established correlates of human resistance to schistosomiasis, while IgG2, IgG4, and IgM are negatively correlated (190–194). IgA to the tegumental protein Sm28GST was also correlated with resistance in one study of human subjects (195). IgE is known to mediate mast cell degranulation, however, paradoxically, mastocytosis was found to correlate with susceptibility to reinfection in one occupationally exposed human schistosomiasis cohort (196). The negative correlation of IgM, IgG4, and IgG2 with human resistance has been attributed to their ability to block IgE and IgG-mediated effector mechanisms of parasite killing *in vitro* (191, 197–199). The factors that stimulate skewing toward production of either protective or blocking antibodies, sometimes to the same targets, are unknown.

An alternative hypothesis for the association of IgE with protection from schistosomiasis has been formulated based on the recent observation that CD23^+^ B cells are associated with resistance in a Kenyan cohort (200). B cells bind parasite-specific IgE through CD23, the low-affinity IgE receptor, and upon encountering parasite antigen, are activated by IgE crosslinking to endocytose the antigen. This mechanism could enable a large population of B cells to present parasite epitopes to T cells, which would in turn activate cognate parasite-specific B cells. The increasing amount of parasite-specific IgE could thus steadily increase the magnitude of the antibody response over the course of several infections, outweighing the immunosuppressive effects of some worm products (201). Such a robust IgE, IgG1, IgG3, and IgA antibody response would perhaps then be capable of destroying larvae and/or adult worms through a combination of the mechanisms discussed above.

T cell-mediated immunity may also play a role in the defense against helminth infection. Mice repeatedly vaccinated with irradiated *S. mansoni* cercaria develop a high level of protection which has been attributed to both Th1 and Th2 mechanisms, including complement activation, CD8^+^ T cell cytotoxicity against schistosomula, and T cells and macrophages trapping schistosomula as they migrate through the lung (202–205). The protection of these mice is dependent on both antibodies and T cells (206–208). The role of Th1 responses in humans is still unclear. In some populations endemic for schistosomiasis and lymphatic filariasis, a mixed Th1/Th2 profile is associated with an effective immune response, whereas in hookworm infection, only Th2 appears to be correlated with resistance (22, 209). In some human populations, resistance to schistosome infection is correlated with increased production of IFNγ by CD4^+^ T cells stimulated with recombinant Sm14 protein and other antigens (210, 211). Polymorphisms in the IL-4 and IFNγ genes have also been associated with resistance levels (212). Thus, while many possible *in vitro* and *in vivo* mechanisms against helminths have been described, it is yet unclear which, if any, of these is implemented by a successful human immune response, and which would be desirable in an anti-helminthic vaccine.

### The role of glycans in adaptive immunity to helminths

Helminths produce an abundance of glycoconjugates that are a rich source of antigens for the immune system of their definitive hosts. For example, the *S. mansoni* cercarial glycocalyx, some of which is shed into the skin during penetration and some of which is retained on the parasite surface, is around 80% carbohydrate by weight (17, 149, 150). In fact, the majority of the human and animal antibody response to schistosomes is directed to glycan antigens (213, 214). Anti-glycan antibodies (αGAbs) are a common feature of helminth infections. It has been challenging to define their role in protection, in large part because, as described above, there is little consensus on the general mechanisms of immunity (anti-glycan or otherwise) that are protective against helminths, with different hosts likely employing different protective mechanisms. This section will highlight the importance of αGAbs and address the continuing challenges to defining their role in helminth infection.

Helminths use specialized mechanisms to invade host organisms and establish a niche in their tissues for long-term survival or to enable passage of eggs out of the host. Helminth glycans are involved in the establishment of such niches, and antibodies to glycans can interfere with this process. The nematode *T. spiralis*, which causes trichinellosis, caps its multi-antennary *N*-glycans with the unique monosaccharide, tyvelose. Monoclonal antibodies to tyvelose are a major component of the natural protection conferred on suckling rat pups by infected dams and protect pups when passively transferred. In epithelial cell culture models, antibodies to tyvelose bind surface glycoproteins of the invading L1 larvae, inhibit migration into the cell layer and interfere with molting (30, 33, 215, 216). This could be how larvae are prevented from colonizing gastrointestinal epithelium in the protective models. While antibodies to tyvelose are protective in the rat model of *T. spiralis* infection, antibodies to PC moieties are not (217). Mucosal antibodies to a carbohydrate antigen of the gastrointestinal nematode *Trichostrongylus colubriformis* also prevent establishment of larvae in the sheep gut (218). In schistosomiasis, eggs must traverse the endothelium and intestinal wall in order to exit the host via stool. Using *in vitro* models of egg attachment to human umbilical vein endothelial cells, antibodies to E-selectin and Lex were shown to decrease adhesion (219). Whether the ability of αGAbs to interfere with host tissue interactions in the models is due to blockage of specific glycan-binding interaction or due to other neutralizing or physically damaging effects on the worms, is unclear. However, interference with invasion or adhesion through blocking surface glycans clearly represents an opportunity to induce protection and/or interfere with pathogenesis.

The antibody effector mechanisms most well known to damage or kill schistosomula *in vitro* are ADCC and complement activation, and αGAbs are capable of both. Pioneering work by the Capron group used a semi-permissive rat model to isolate an IgG2a called IPLSm1. The antibody killed schistosomula *in vitro* via eosinophil-mediated ADCC and passively transferred resistance to naïve rats (171). IPLSm1 targeted a 38-kDa surface glycoprotein which was also recognized by infected monkey and human sera, and was cross reactive with Keyhole Limpet Hemocyanin (KLH) glycans (220, 221). Our present knowledge of KLH and schistosome cross-reactive glycans supports the hypothesis that IPLSm1 targeted the FLDNF glycan (49, 50) (Table 1). The 38-kDa antigen was also used to develop an anti-idiotype vaccine, which conferred 50–80% protection to rats and induced antibodies that mediated ADCC (222). Mice also develop abundant antibodies to LDN-based glycans, including IgE, IgG1, and IgG3 (but not IgG2) to LDNF, indicative of a skewing toward Th2-type antibody effector mechanisms such as ADCC (223, 224). A murine IgM to LDN isolated by our group mediates complement killing of schistosomula *in vitro* (122). The Harn group isolated three murine αGAbs, two of which, an IgM against the Lex antigen and an IgG2b against an unknown carbohydrate antigen, were protective and mediated *in vitro* complement killing, and an IgG3 that was not (61, 225).

Adaptive immunity to glycans may also be involved in aspects of helminth pathogenesis. LN- and LDN-coated beads induce schistosomiasis-like granulomas in murine livers. It is unclear whether this model works through adaptive or innate mechanisms, but fucosylated glycans known to bind C-type lectins did not induce granulomas (226, 227). The anti-Lex antibodies induced by schistosomes are cytolytic to human myeloid cell lines. These antibodies could potentially be responsible for mild neutropenia seen in infected humans, or for killing of schistosomula (63).

Antibodies generated by mammalian hosts to helminth glycans are not only abundant but highly specific. Schistosomes, for example, present the same glycan epitope in a variety of structural contexts, such as on *N*- and *O*-glycans, or as single or multibranched glycans, as diagrammed in Table 1. The structural presentation of such epitopes as Lex and LDNF can vary among schistosome life stages, localization, and sexes (48, 228, 229). Data from our lab and others have demonstrated that monoclonal antibodies and sera from infected hosts can discriminate against very similar epitopes, such as the monomeric, biantennary *N*-glycan, and multimeric forms of the Lex or LDNF trisaccharide epitopes (60, 228). Given that some of these structural variants are somewhat similar to mammalian glycans, this high level of specificity could be crucial to developing an effective parasite-specific antibody response. Anti-schistosomal monoclonal antibodies with well-defined glycan specificity can be used to isolate parasite glycoconjugates and potentially identify novel vaccine targets including both glycan and protein epitopes (60). We and others are developing the Glycomics tools that will help us to better define the specificity of the αGAbs against helminths (230–232).

Whether human resistance to helminth infection is mediated by αGAbs is a fascinating but complex question, which has only been addressed in a handful of studies examining correlative evidence. *S. mansoni*-exposed humans and non-human primates make antibodies to glycan epitopes with fucosylation patterns unique to schistosomes such as FLDN and LDN-DF (233, 234). One group observed that a Kenyan population showed decreases in IgG1 to FLDN and LDN-DF, and increases in IgM to LDN-DF and LDNF, over the course of 2 years after migrating from a non-endemic to schistosomiasis-endemic area; the same associations were seen with increasing age in the schistosomiasis-endemic resident population (234). Levels of IgE to worm glycolipids pre-praziquantel treatment were inversely correlated with egg burden 2 years after treatment in another population (235). Using shotgun glycan microarrays made from the intra-mammalian stages of schistosomes, other investigators have found that *S. mansoni*-infected adults make IgG and IgM to several fucosylated glycan epitopes, and that children have modestly higher titers than adults to most glycans (230, 236). Collectively, these studies are difficult to interpret, due to the challenges of identifying human populations that truly show variable resistance and susceptibility (mechanisms of which likely differ among populations), the difficulty of obtaining glycan preparations that are both pure and accurately mimic the mode of presentation by the parasite, and the differential significance of antibody isotypes and sub-isotypes in human resistance. Further studies are needed to strengthen these correlations and more directly examine the role of αGAbs in protection from schistosomiasis and other human helminth infections.

Other reports have indicated that antibodies to glycans can be non-protective or even block the development of resistance to helminths. *Heligmosomoides polygyrus*, a well-studied mouse model of gastrointestinal nematode infection, elicits a non-protective immunodominant response to an O-linked glycan on VAL antigens (237). Following isolation of a protective IgG2a against *S. mansoni* 38-kDa antigen mentioned above, a second antibody, an IgG2c that targeted the same glycan, was isolated from infected rats. The IgG2c blocked the protective effect of the IgG2a *in vitro* and *in vivo*, which may be why a response to this epitope was correlated with infection in humans but not with resistance (197). It had earlier been hypothesized, based on results from a complex series of experiments on chronically infected and radiation-attenuated cercariae vaccinated mouse sera, that levels of antibody to parasite surface antigens is not simply correlated with protection. Protection may instead depend on a particular balance of blocking and protective antibodies, possibly against the same antigens (238). Clearly such counteractive effects of antibodies to glycan antigens should be explored in more detail.

The lesson of all of these studies is that helminth glycans, like protein epitopes, can induce both protective and non-protective antibodies. Rather than viewing glycans as a class of targets and asking, “is their role protective or subversive?” we should continue to identify particular anti-glycan specificities and isotypes that can afford protection, design experiments to directly test their role, and develop technologies to better understand which structural presentations and innate cues are required to incite production of protective versus non-protective antibodies.

### Applications of helminth glycans to diagnosis and vaccination

It has long been known that helminths synthesize unique glycan structures, which are targeted by the adaptive immune response in natural infection; however, this rich collection of antigens has yet to translate into molecular targets for diagnostics and vaccines. This section will emphasize research on how control of helminth infection can be improved by exploiting glycans as novel diagnostic and vaccine targets.

Treatment of helminth infections currently relies on chemotherapeutics such as albendazole and praziquantel (6, 239). Prevalence in some areas is so high that mass drug administration (MDA) has been implemented for school-age children. Chemotherapy significantly decreases worm burden and morbidity but is not always curative, and its effectiveness varies depending on the worm life stage. Single-dose cure rates range from 15 to 72% in various helminth infections (239). However, the effectiveness of MDA in controlling transmission and reducing morbidity is difficult to determine, because traditional diagnostic methods are laborious and insufficient to detect low-level infection or track variations in worm burden (240, 241). The “gold standard” for diagnosis of helminth infection continues to be microscopic examination of stool or urine samples for eggs. However, eggs are not consistently shed into feces and urine. Despite improvements in the sensitivity and ease-of-use of these tests, stool samples are still difficult to obtain in the field, often yield false negatives due to temporal variation in egg-laying, and differentiation of the type of helminth eggs in stool requires skilled laboratory technicians (239–242).

Commercial ELISA-based detection kits are available for diagnosis of some parasites including malaria (*Plasmodium* species), cryptosporidiosis, and giardia in stool, urine, or serum samples. For helminths causing schistosomiasis, filariasis, and trichinellosis, antibody-based tests are available from commercial sources or by special request from the CDC but are not widely used in endemic areas (9, 243). Antibody tests are generally sensitive, but they suffer several drawbacks, such as inability to differentiate between active (acute or chronic) and past infections, cross-reactivity among multiple helminth species, and difficulty of performance in the field (242, 243).

Recent studies have uncovered a new set of potential diagnostic antigens, found in serum and urine, for schistosomiasis and other helminths. Carbohydrate-based antigens and αGAbs are promising tools given that they are chemically stable, specific to particular helminth species, vary with stage of infection, and are expressed both on worm surfaces and in secreted products. Several glycan-based detection methods are now in the pipeline for schistosomiasis (244, 245). A point-of-care urine dipstick test for the schistosome excreted circulating cathodic antigen (CCA) (Table 1), whose antigenicity is due to Lex repeats, is now commercially available (245, 246). It is easier to perform in the field and has higher sensitivity than a single Kato-Katz smear, and it can detect prepatent infections in very young children (245, 247, 248). Additionally, a test for the other well-studied circulating schistosome glycan antigen, circulating anodic antigen (CAA), which is excreted by adult worms into urine and serum, has recently been adapted for field use with promising results. The test is highly sensitive and can detect just a few worm pairs (249). These and other novel diagnostic tests are ready for rigorous comparison in the field and are likely to change the face of schistosomiasis diagnostics in upcoming years. Another epitope, apparently unique to schistosomes, is DF-LDN-DF, which forms the epitope for the monoclonal antibody 114-4D12. This antibody can be used to isolate free urinary glycans for detection by mass spectrometry, and to identify the DF-LDN-DF on egg glycoproteins from the blood or urine via ELISA (243, 250, 251).

Molecular detection of trichinellosis identifies antibodies to the TSL-1 glycoprotein, of which β-tyvelose is the immunodominant epitope. Synthetic tyvelose outperformed worm ES antigens in detection of these antibodies via ELISA (9, 72, 252). The cestode *Echinococcus multilocularis*, which causes rare but serious infection in humans, is detected by ultrasonography and antibodies to the Em2 glycoprotein (9, 253). Recently, it was shown that the immunodominant Em2 epitope is a unique O-linked glycan capping structure, Galpα1-4Gal, and that antibodies to this structure were highly sensitive and specific for detection of infected patient sera via ELISA (254, 255). The same group has also identified a novel glycoprotein for detection of *E. multilocularis* infection in dogs, which may be an important source of human acquisition (253).

Modern glycan microarray technology is also being used to identify new glycan candidates for diagnosis of helminths. Studies have shown that LDNF, which is more easily produced in the lab than tyvelose, is also a sensitive indicator of *T. spiralis* infection (256). Similarly, results from glycan microarray analyses have shown that the sheep nematode *H. contortus* possesses Galα1-3GalNAc, which is antigenic and uncommon among nematodes and trematodes (257). These new microarray technologies have the promising ability to screen a single sample for antibodies to multiple glycans from different helminth species, many of which are co-endemic, in a microscale assay. Thus, glycan arrays have enormous potential to define the diagnostic antigens of the future.

Development of resistance to anti-helminthics, especially in the face of MDA, has long been of concern. Reduced susceptibility to praziquantel has been reported in some human schistosomiasis-endemic areas, and it is possible to generate resistant schistosomes in the lab (258). Only one new anti-helminthic, tribendimidine, has become available in the last 30 years (259, 260). However, its mechanism is similar to two existing anti-helminthics, and little research is taking place to discover novel mechanisms and drug targets (6, 261). Donations of such drugs are currently meeting only 5 and 49% of the global need for schistosomiasis and hookworms, respectively, and these drugs do not interrupt the chain of transmission, owing to variable efficacy rates, animal reservoirs, and frequent re-infections in children (3, 5, 7, 242). Clearly, vaccines that expedite the development of immunity are a much-needed intervention in control of helminths.

Animal models of vaccine-induced immunity to helminths have used attenuated parasites and worm lysates or other worm products. Due to the difficulty of maintaining a complex life cycle in large scale, and the danger associated with manufacturing this type of vaccine, it is unlikely to be a practical solution. Modern vaccine development for parasitic helminths has focused on recombinant proteins but not on glycoproteins, which represent the major targeted antigens of infection. In the mid-1990s, six *S. mansoni* proteins, studied in various labs, were chosen by the WHO to undergo independent laboratory testing. None of these reached the required 40% effectiveness required to move past animal testing (262). Two candidates have more recently reached the clinical phase. Bilhvax (Sh28-GST) has progressed through phase I, II, and III trials, however, there has been a more than 10-year delay in publishing the results (263). Another candidate schistosomiasis vaccine, Sm14, may enter clinical trials this year, and at least two more candidates are progressing through the pre-clinical pipeline (264). The *N. americanus* protein ASP2 was clinically tested for prevention of hookworm, but recipients of the vaccine developed hives (265, 266). Currently two more hookworm candidates, GST1 and APR1, are being developed and clinical testing for GST1 should start soon (8, 265, 267). These studies highlight the difficulty of identifying effective targets and inducing the proper character of immune response for helminth vaccines. Modern methods of producing recombinant glycoproteins may allow future targeting of specific glycoprotein antigens for vaccine studies.

Localization of target proteins may be one of the problems with early vaccine candidates for schistosomiasis. Several of the protein candidates were later identified in the worm tegument, but only one was found in apical membrane preparations (14, 268). A newer strategy is to use proteomic studies to identify protein candidates which are exposed on the worms’ surface, accessible to immune effectors, and vital for worm functions such as membrane assembly and blood feeding (8, 269, 270). One of these studies used biotinylation reagents to label accessible adult *S. mansoni* tegument proteins. Only a small subset of proteins was identified, suggesting that many surface proteins are shielded from immune attack by the glycan and lipid-rich membranocalyx (8, 270). In light of the difficulties faced in developing recombinant protein candidates as schistosome vaccines, we suggest taking advantage of the rich collection of non-protein antigens surrounding vulnerable stages of the worms.

Given the rapid turnover of helminth surface antigens, variation in their expression among life stages, a successful vaccine may need to target more than one epitope. Glycan epitopes offer the advantage of being densely distributed on numerous glycoconjugates on the parasite surface, and expressed throughout multiple life stages. The schistosome is a well-characterized demonstration of this observation. Our group has shown that LDN and LDNF are expressed on the surface of *S. mansoni* cercariae, schistosomula, and adult worms as well as in SEA (122), and that LDNF is expressed on all three major schistosome species (223). Other fucosylated variants which are not shared by mammalian hosts, such as LDN-DF and FLDNF, have been localized to eggs, cercariae, adult gut, and tegument, and appear on numerous distinct glycoproteins and glycolipids as detected by ELISA, Western blotting, and immunofluorescence of whole parasites and parasite sections (20, 271). An additional advantage is that because glycan structure is not linearly encoded in the genome, selective pressure is less likely to result in the escape of glycosylation mutants than is the case for proteins.

Vaccination experiments have also demonstrated that eukaryotic glycoconjugates are viable vaccine targets. Vaccination of lambs with alhydrogel-adjuvant excreted/secreted products of the nematode *H. contortus* conferred a high level of protection which was correlated with IgG antibodies to LDNF and Galα1-3GalNAc (257, 272). Other studies that used natively purified activation-associated secreted proteins (ASPs) from the cattle nematode *O. ostertagi* also afforded protection, and showed that the recombinantly produced ASPs from *E. coli* were unable to induce protection or any antibodies to native ASPs (273). Hybrid-type *N*-glycan structures were characterized on the native protein and, while antisera were not directly reactive with the glycan structures, it was hypothesized that they were necessary for proper folding of the native antigen. Another approach taken was to explore an anti-idiotype vaccine, which was found to be protective in rats and to generate immunity to a 38-kDa glycoprotein antigen mentioned above (222). A vaccine against a *P. falciparum* glycosylphosphatidylinositol (GPI) induced IgG that was able to neutralize parasite pathogenesis *in vitro* (274).

Further studies are needed to better define the glycan antigen structures of helminths, to develop novel methods of producing and presenting eukaryotic glycans in an immunogenic fashion, and to discover the glycosyltransferases necessary to generate the worm glycan structures that are foreign to mammals. The central role of glycans in adaptive immunity to helminths and these early studies into their protective capacity indicates that, with further innovation, glycan-based diagnostics and vaccines may be an important intervention in the control of helminth infection.

## Glycan Interactions with Intermediate Hosts

As previously stated, schistosomes synthesize a complex array of glycan structures on both membrane and secreted glycoconjugates. Many of these glycans have been found to be potent antigens in vertebrate hosts, but their roles in snail infections are poorly understood (275–277). Emerging evidence suggests that schistosome glycoconjugates play a pivotal role in both cellular and humoral immune interactions between their molluscan intermediate hosts and the infecting larval stages (277–279).

There appears to be a role for fucosylated carbohydrate epitopes expressed by larval and adult schistosomes in parasite evasion in intermediate and definitive hosts (275, 280, 281). During invasion of the snail body, the miracidia penetrate the epithelium allowing for direct interaction of the snail tissues with the miracidial glycocalyx. The carbohydrate epitopes present on the surface of the miracidium during this time may be of prime importance during the invasion process. Recently, it was shown that *B. glabrata* synthesizes a broad battery of *N*-glycans on multiple glycoproteins comprising at least two carbohydrate determinants that cross-react with glycoconjugates from *S. mansoni* eggs (282).

It is well known that *S. mansoni* glycan expression is developmentally and stage-specifically regulated, but until recently, the glycan epitopes expressed in miracidia and sporocysts were largely unknown. Using a mass spectrometry approach for glycomic profiling, Hokke et al. found evidence for expression of multifucosylated, LDN-terminating di- and tri-antennary structures, as well as the presence of the truncated trimannosyl and core-xylosylated/core-α-1,3-fucosylated *N*-glycans in miracidia (283). Lehr et al. demonstrated the surface expression of FLDN, FLDNF, LDNF, and LDN-DF in miracidia and the presence of these, as well as non-fucosylated LDN, and Lex glycans in secondary sporocysts (275, 282, 283). Alpha-1,3-fucosylated LDN structures (FLDN, FLDNF, LDNF) are prominently expressed on the larval surface and amongst glycoproteins released during larval transformation and early sporocyst development. This stage-specific expression implies a role for these glycans in snail–schistosome interactions. Also, sharing of specific glycans FLDN and trimannosyl *N*-glycans with *B. glabrata* suggests an evolutionary convergence of carbohydrate expression between schistosomes and their snail host (275).

Larval glycans and/or their associated glycoconjugates might also be serving as PAMPs that interact with lectin-like PRRs (284). PRRs, such as Toll receptors, C-type lectins, galectins, nucleic acid-sensing receptors, and the intracellular nucleotide-binding oligomerization domain (NOD)-like receptors (NLRs) occur both extracellularly and intracellularly, with the galectins notably found in both the cytoplasm and extracellularly (285). In terms of glycoconjugates in helminth infections and their interactions with all types of PRRs, little is known, and the most well-studied interactions involve C-type lectins and galectins. There is also evidence that glycans may be important in the intermediate hosts’ innate immunity and PRR recognition and may involve novel PRRs. During their development in the molluscan intermediate host, *S. mansoni* sporocysts release excretory/secretory glycoproteins that bind to lectin PRRs on the surface of the snail host hemocytes and are believed to modulate the ability of the hemocytes to interact with the developing larvae (279). The binding of glycoconjugates to *B. glabrata* hemocyte lectins can trigger the generation of parasite-killing reactive oxygen species, thereby mediating innate immune responses to invading miracidia (10, 275, 277, 280, 281, 284).

Glycans may also be the targets of humoral immune responses mounted by the molluscan hosts against larval infection. *B. glabrata* snails respond to infection by secreting humoral factors into their hemolymph that bind and precipitate larval excretory/secretory antigens. These factors contain N- and C-terminal domains with similarities to immunoglobulin super-family proteins and fibrinogen, respectively, and are called fibrinogen-related proteins (FREPs) (277, 284, 286). To counteract FREPs, developing primary sporocysts envelope themselves in a glycan-rich environment comprised mainly of glycoproteins and other glycoconjugates referred to as larval transformation products (LTPs) (284, 287). LTP glycoconjugates released during transformation are able to alter patterns of shared glycan epitopes by either binding and blocking, or by exposing them. This is a possible mechanism by which molecules released from early developing larvae may impact initial immune interactions at the host-parasite interface and shows the potent immune modulating effects of LTPs (284, 288).

## Conclusion and Future Perspectives

Molecular insights into the innate and adaptive immune responses to glycoconjugates of parasitic helminths are providing new directions for developing diagnostics, therapeutics, and potential vaccines toward these organisms. Developing evidence indicates that parasitic helminths utilize a wide variety of glycosylated molecules to successfully infect their vertebrate and often invertebrate hosts. The parasite glycans are characterized by their complex structures that are often multifucosylated and rich in unusual monosaccharides and modifications, making them strong targets for adaptive immune responses. Such unusual glycans also demonstrate strong recognition and signaling by DCs and MΦs, through lectins, TLRs and CLRs, and other antigen-processing cells that serve to limit inflammation and promote parasite survival. The cross talk that occurs from these glycan-dependent signals is important in initiation of the adaptive immune response, but could also contribute to the overall polarization of immunosuppressive responses to the parasite infections. Many glycoconjugates of parasites are potent immune modulators which have the potential to be channeled into effective immunoregulatory therapies with potential for treating multiple chronic inflammatory diseases, such as MS or Crohn’s disease. While glycans are targets in natural infections, much remains to be learned about the expression and functions of parasite-derived glycans, and their potential role in resistance to infection. While some glycans are useful in diagnostics and monitoring, none of the specific glycans of these parasites has yet been translated into molecular targets for vaccines.

Some of the key questions that need to be addressed in helminth glyco-immunology are: what is the full range of unique helminth glycans and how is their expression on glycoconjugates regulated? What is the full repertoire of glycan-binding proteins or receptors on host cells that function to respond to helminth glycans? Which glycans are responsible for the immunosuppressive effects of helminth products? What signaling pathways mediate the complex cross-talk among CLRs and other PRRs? What are the vaccine design considerations for utilizing parasite glycan antigens, which are structurally distinct from repeating bacterial polysaccharide antigens? Which anti-glycan antibody isotypes/arms of effector immunity are protective in helminth infection? Can glycans be used as diagnostics to differentiate among co-endemic helminth infections and active versus cured infections? And could glycan-based interactions with intermediate hosts be exploited for transmission control? Given the growing realization that the parasite glycome is active in pathogenesis and resistance, it will be exciting to see the coming results from future research in this key area of biomedical importance worldwide.

## Conflict of Interest Statement

The authors declare that the research was conducted in the absence of any commercial or financial relationships that could be construed as a potential conflict of interest.
